# Motivational traits: An objective behavioral test using a computer game

**DOI:** 10.3389/fpsyg.2022.812918

**Published:** 2022-10-18

**Authors:** Daniel Fernández-Avilés, Angélica de Antonio, Elena Villalba-Mora

**Affiliations:** ^1^Madrid HCI Lab, Research Group on Human–Computer Interaction and Advanced Interactive Systems, Universidad Politécnica de Madrid (UPM), Madrid, Spain; ^2^Center for Biomedical Technology (CTB), Universidad Politécnica de Madrid (UPM), Madrid, Spain; ^3^Centro de Investigación Biomédica en Red en Bioingeniería, Biomateriales y Nanomedicina (CIBER-BBN), Madrid, Spain

**Keywords:** motivational trait, objective behavioral test, computer game, motivation modeling, personality, game-based assessment

## Abstract

An objective behavioral test for motivational traits has been developed taking as a reference the structure of the Motivational Trait Questionnaire, which is a validated self-report questionnaire to measure motivational traits in the population. The objective test consists of a computer game, which makes it possible to evaluate a person’s motivational traits and to display them on the same scale as the Motivational Trait Questionnaire. To evaluate the validity of the results obtained in the measurement of motivational traits using the objective behavioral test, a study was carried out with 31 participants whose motivational traits were evaluated using the two methods, and the results obtained were compared to find out whether the two forms of assessment can be considered equivalent. Statistical tests support the high degree of similarity of the results, concluding that the objective behavioral test can be a useful instrument to assess motivational traits as an alternative to the self-report questionnaire. Despite the increasing popularity of game-based assessment of personality traits, this is the first time a game has been designed for the assessment of motivational traits. Being able to obtain a model of the user’s motivational traits without having to rely on a questionnaire opens the possibility to build software applications that adapt to the user’s motivational profile, providing them with the kind of motivational support that best fits their needs.

## Introduction

Our research aims to design mechanisms that allow a computer system to model a user as completely as possible, from the motivational point of view, and in such a way that, as far as possible, this modeling process occurs automatically and hidden from the user, through the analysis of their interaction with the system. Motivational modeling is intended to cover in the first place the most stable and defining aspects of the person, corresponding to the motivational traits. Taking an existing questionnaire for the assessment of motivational traits as a reference, we have designed an objective behavioral test in the form of a game, where the player is faced to specific situations in which the behavioral decision made will be interpreted as evidence of certain motivational traits. The self-report questionnaire we have taken as a reference is the Motivational Trait Questionnaire (MTQ) (Kanfer and Ackerman, 2000).

It is important to note that the game will be used merely as an instrument to infer motivational traits. The relationship between games and motivation has been extensively studied, with the concept of gamification emerging to acknowledge the motivational power of games. Some researchers (Lewis et al., 2012) have aimed to provide game designers with game design patterns that implement motivational techniques to motivate player behavior. Others (Leitão et al., 2022) have investigated the motivational power of different game elements in a gamified learning environment. Our goal, however, is not to use the game as a motivational tool, but as a scenario where motivational traits determine the user’s behavior, thus facilitating the inference of those traits from the observed behavior.

The interplay between games and personality has also been explored. Recent research (Tasnim and Eishita, 2021) analyzes the differential impact of personality traits on the gameplay experience. More directly related to the research reported here is the use of game-based assessments of personality traits (full games used to assess specific constructs), which is becoming increasingly available and popular (Weidner and Short, 2019; Wu et al., 2022). In some cases, rather than developing an entire game, individual mechanics or dynamics from games are identified as targets for inclusion in an assessment. In the case of gameful design, the assessment is created with such a goal, whereas in gamification, an existing assessment is modified to meet it (Harman and Brown, 2021; Landers and Sanchez, 2022) gamefully designed narrative-based measures of personality. A representative of gamification is (Landers and Collmus, 2021), who converted existing multiple-choice personality measure items into a story version in which each item of the original measure became a situational judgment item in the gamified version.

Extracting user models from user interaction with a software application has been extensively researched in the field of intelligent and adaptive learning systems (Virvou et al., 2012). Although this kind of student models mainly concentrate on representing the student’s knowledge, they may also include affective, motivational, and other psychological states that are inferred from performance data during the course of learning. Adaptation to the motivational state of the student has been identified as a key element in adaptive educational systems (Orji and Vassileva, 2021), but automatic inference of the more stable motivational traits has not been tackled yet in student models. Whenever motivation as a personality characteristic has been considered, it has been evaluated *via* conventional self-report instruments (Wighting et al., 2008).

Once our game-based objective behavioral test was designed, it was relevant to investigate the possible degree of similarity between the results obtained from the self-report questionnaire and the objective behavioral test. If the degree of similarity is high, both tests could be considered equivalent with a margin of error acceptable to researchers. Once the data was obtained with both procedures, we analyzed the possible correspondences and discrepancies between the two procedures and the reasons for them.

This paper is organized as follows. In Section II, the different sources of data for personality assessment are discussed, highlighting the advantages and requirements for objective tests. Section III explains the design of the computer game-based objective test developed: the description of the game, the motivational metric that categorizes the participants, the developed software architecture and the technologies needed for its implementation. The materials and method applied in the evaluation of the objective test are explained in Section IV. Section V shows the results obtained. The discussion is carried out in Section VI, and Section VII concludes this paper.

## Assessment of motivational traits

### Sources of data: Self-report versus objective test

Of the three different sources of data (Q data, L data, and T data) defined by Cattell (1965) in personality assessment, we will focus on Q data (questionnaire) and T data (objective test). Q data are obtained through responses to questionnaires or self-report inventories. Catell distinguishes between two types of Q data, the one he considers to be more valid, in which item responses have been shown to correlate with actual behavior, and Q’ data that are based on unverified introspections. The third source of data is the T data, which are obtained through behavioral tests in a defined situation, in which the purpose of the test must be hidden from the subject in order to analyze their behavioral response to that situation.

We must highlight several important problems about Q data, such as the possible lack of sincerity of the participants, the self-image they have in relation to an item (which may be true for them and different for the rest), or the level of understanding of the participants since, if they do not understand the questions, the answers will be meaningless.

Regarding the T data, several requirements have been defined to perform valid and reliable personality assessments using a computer-based objective test (Cattell, 1965; Cattell and Kline, 1977; Ribes and Sánchez, 1992; Santacreu et al., 2006; Ribes, 2009; Romero Velázquez et al., 2019).

(a) Test participants must be capable of performing the tasks they are asked to perform, so the tasks should not require a high level of skill or dexterity.
(b)Test takers should be motivated to perform the tasks requested in the test.
(c) Test takers must understand the purpose of the tasks and be able to follow the instructions correctly. Therefore, the instructions must be clear.
(d)The test instructions should not influence or direct the behavior of the participants throughout the test. It is important that participants apply their behavioral strategies naturally.
(e) The test should not provide feedback on the personality variable that the researchers wish to assess. In this type of test, the participant is unaware of the relationship between their response and the personality characteristic that is intended to be measured or assessed.

It can be problematic to try to correlate these two sources of data, since there is not always a strong relationship between the demonstrated behavior of a person facing a concrete situation presented to them and the behavior self-reported by the same person when imagining that situation. Several studies (Skinner and Howart, 1973; Cattell and Kline, 1977) have found a relative lack of correlation between data obtained by questionnaires and data obtained by objective tests.

Taking into account the advantages of objective tests for the assessment of personality traits, compared to questionnaires, we decided to face the design of a computer-based objective test capable of assessing motivational traits based on the behavior of the participant.

### The motivational trait questionnaire

Our first step was an analysis of questionnaires that had been proposed for the assessment of a person’s motivational traits and the selection of the one to be used as a reference to compare the results obtained in our objective test. As a result of this analysis, Kanfer and Heggestad’s MTQ questionnaire (Kanfer and Heggestad, 1997) was selected.

Kanfer and Heggestad’s research attempts to integrate advances in the theory and practice of personality assessment with those of motivation/self-regulation. They proposed a framework (Motivational Traits and Skills framework, MTS) that attempts to integrate and organize all individual differences related to motivation and provide a cohesive view of the area.

They suggest the usefulness of differentiating between motivational traits and motivational skills. Motivational traits are defined as stable, cross-situational individual differences in preferences related to approach and avoidance, in the investment of goal-directed effort. On the contrary, motivational skills are defined as integrated self-regulatory competencies that are put into practice during an attempt to achieve a goal.

Kanfer and Heggestad (1997) first identified the traits potentially relevant to motivation from the analysis of different research trends and used Trait Construct Clustering (Snow et al., 1996) to organize the domain, resulting in two major motivational trait complexes, Achievement and Anxiety. While the Achievement complex includes traits that are characterized by approach tendencies, the Anxiety complex traits are characterized by avoidance tendencies.

Within the Achievement complex, there are three motivational traits:

- Personal Mastery (PM): An individual with a high value for this trait defines excellence standards in terms of personal improvement and persists in attempting to reach those standards despite frustrations and difficulties. They generally show a preference for tasks that challenge their skills and abilities. They are competitive with themselves and always try to be the best they can be.
- Competitive Excellence (CE): An individual high in this trait adopts normative standards of excellence. Absolute quality in performance is not of great importance to these individuals because they define success in relation to others; what matters is that their performance is superior to that of others. They are highly competitive individuals who try to transform non-competitive situations into competitive ones. Additionally, they have a strong desire to be respected by others for their performance.
- Hard Work (HW): A trait not initially identified but later included. An individual with a high value on this trait tends to invest great effort to complete tasks, regardless of their level of intrinsic enjoyment with the task. They are hard workers and diligent. They have a strong desire to be busy and find it difficult to relax and do nothing.

Within the Anxiety complex, two motivational traits are found:

- Failure Avoidance (FA): An individual with a high value for this trait actively tries to avoid, whenever possible, achievement-oriented situations because of the anxiety caused by the possibility of failure.
- Achievement Anxiety (AA): Reflects the tendency to experience anxiety responses to achievement situations (i.e., where there is a possibility of failure). It is broader than Assessment Anxiety, which refers only to assessment situations in the academic context.

Kanfer and Heggestad proposed a questionnaire that initially contained 283 items, which in turn were organized into several content facets within each trait. However, although the results of the factor analysis of the facet scales proposed in the MTQ questionnaire provided evidence for the multidimensional structure of motivational traits, only three factors emerged instead of the five initially proposed: Personal Mastery (combining Personal Mastery and Hard Work), Competitive Excellence, and Achievement Anxiety (combining Achievement Anxiety and Failure Avoidance) (Heggestad and Kanfer, 2000). Furthermore, evidence of construct validity for MTQ traits was found through correlations between these factors and existing measures.

Although there is little empirical evidence on the predictive validity of MTQ, relationships between motivational traits and task performance, mediated by goal setting mechanisms and self-efficacy, have been found to exist.

There are currently two versions of the questionnaire, a long version MTQ-LONG with 82 items, and a short version (MTQ-S, Motivational Traits Questionnaire Short Form) with 48 items (Kanfer and Ackerman, 2000). The latter measures three dimensions of motivational traits: Personal Mastery (PM), Competitive Excellence (CE), and Motivation Related Anxiety (MRA). Within each dimension, there are two subscales. The long questionnaire adds one more dimension, Failure Avoidance (FA), and distributes its 82 items in 9 subscales. Each item of both questionnaires is answered on a Likert scale ranging from 1 (Very UNCERTAIN about me) to 6 (Very CERTAIN about me). This questionnaire is not publicly available, therefore, we asked permission to use it, and the authors granted it.

In the present study, we used the 82-item long questionnaire with four motivational dimensions. However, the Motivation Related Anxiety (MRA) dimension has been excluded from the implementation of the objective test because all items corresponding to this motivational dimension are related to physiological reactions of the human body produced by possible stressful or overwhelming situations, such as stomachache or cold sweat. Due to the impossibility of our computer game to capture this type of physiological reactions, it has been decided to exclude this motivational dimension.

## Design of the objective behavioral test for the assessment of motivational traits

Given that playing is an intrinsically motivating activity, it was decided that the objective test to be designed should take the form of a computer game to comply with requirement (b) above, which we have called the Motivational Traits – Game-based Objective Test (MT-GOT). In the design of the game, the first step consisted of an analysis of the MTQ items, to extract the set of specific situations or experiences that the user must imagine when performing the MTQ, in order to replicate them as closely as possible. In this way, the user will not be asked to imagine the proposed situation, but to face and react to it while playing. The different situations that have been extracted from the analysis of the MTQ dimensions and are considered in the design of the game are listed below.

Determination (D):

- Situations in which the user can advance through many attempts, and that means an overexertion (costly).
- Situations in which the user can advance easily and without much effort (easy).

Mastery goals (MG):

- Situations in which the user can set themselves marks or challenges to improve.
- Situations in which the user can improve their own records to improve.
- Situations in which users can challenge themselves.

Desire to learn (DL):

- Situations in which the user can learn a skill or knowledge in different stages.
- Situations in which the user can consult and search for information.
- Situations in which the user does not need to read all the information in the system to successfully complete the objectives.
- Situations in which the user does not need to make an effort to obtain a minimum score or pass a level.

Other referenced goals (ORG):

- Situations in which the user can compare their performance with other users.
- Situations in which the user can show whether they care about the opinion of other users about them.
- Situations in which the user can show their satisfaction with their own performance by being compared with a user who does better and worse than them.

Competition search (CS):

- Situations in which the user can choose whether they want to compete or cooperate with other users.
- Situations in which the user must compete and cooperate with other users on equal terms.

Failure avoidance (FA):

- Situations in which the user can decide the risk (easy, medium, and hard) to be taken in their actions.
- Situations in which the user fails, and the system offers the option to try again.

### Description of the game

The game is implemented as a 3D virtual space in which the player finds several tables distributed in a room, which give access to the different modes and functionalities of the game. The player will have an avatar with which to move around the scenario at will. In addition to the game tables, there are some hidden minigames or surprises, like “easter eggs,” that the user will only be able to discover by taking the time to explore the scenario.

After logging into the game, the system will show the user a tutorial, to comply with requirement (c) above, that provides information about the elements of the graphical interface, how to move through the virtual environment and how to interact with it, and describes the objective of the game, the game rules, available options, how to successfully complete the game, and the minimum requirements to be met. As the tutorial presents information to the user, they will be given the opportunity to advance in the normal sequence of information of the current block, to skip the rest of the information in the block, or to request even more complementary information. Thus, it will be up to the user to decide the amount of information to be received (reduced, medium, or exhaustive).

**Figure 1:**
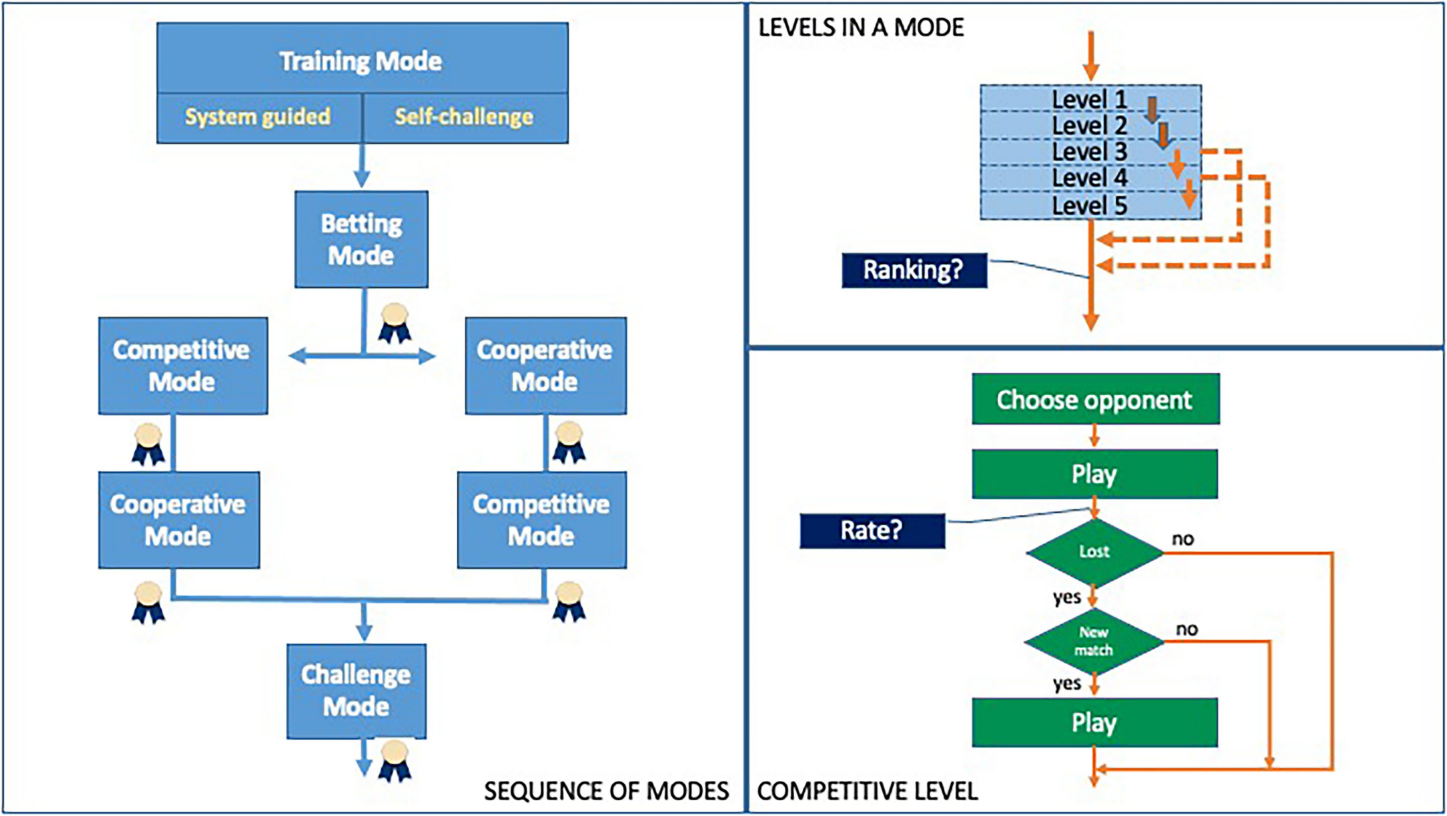
Overview of the MT-GOT structure.

To complete the game, the user must complete five game modes, each of which, in turn, includes five levels, so the user must pass at least three levels of a mode to complete it. When the user successfully completes a level, they will receive points and coins and advance to the next level. When a user completes a mode to the maximum level, that is, level 5, they will receive a reward and will be given the opportunity to appear or not in the ranking corresponding to that mode. The first mode to be completed is the Training Mode, followed by the Betting Mode. Once this mode is completed, two more modes will be unlocked: the Competitive mode and the Cooperative mode. Finally, once these two modes have been completed, the Challenge Mode will be unlocked (see Figure 1).

**Figure 2:**
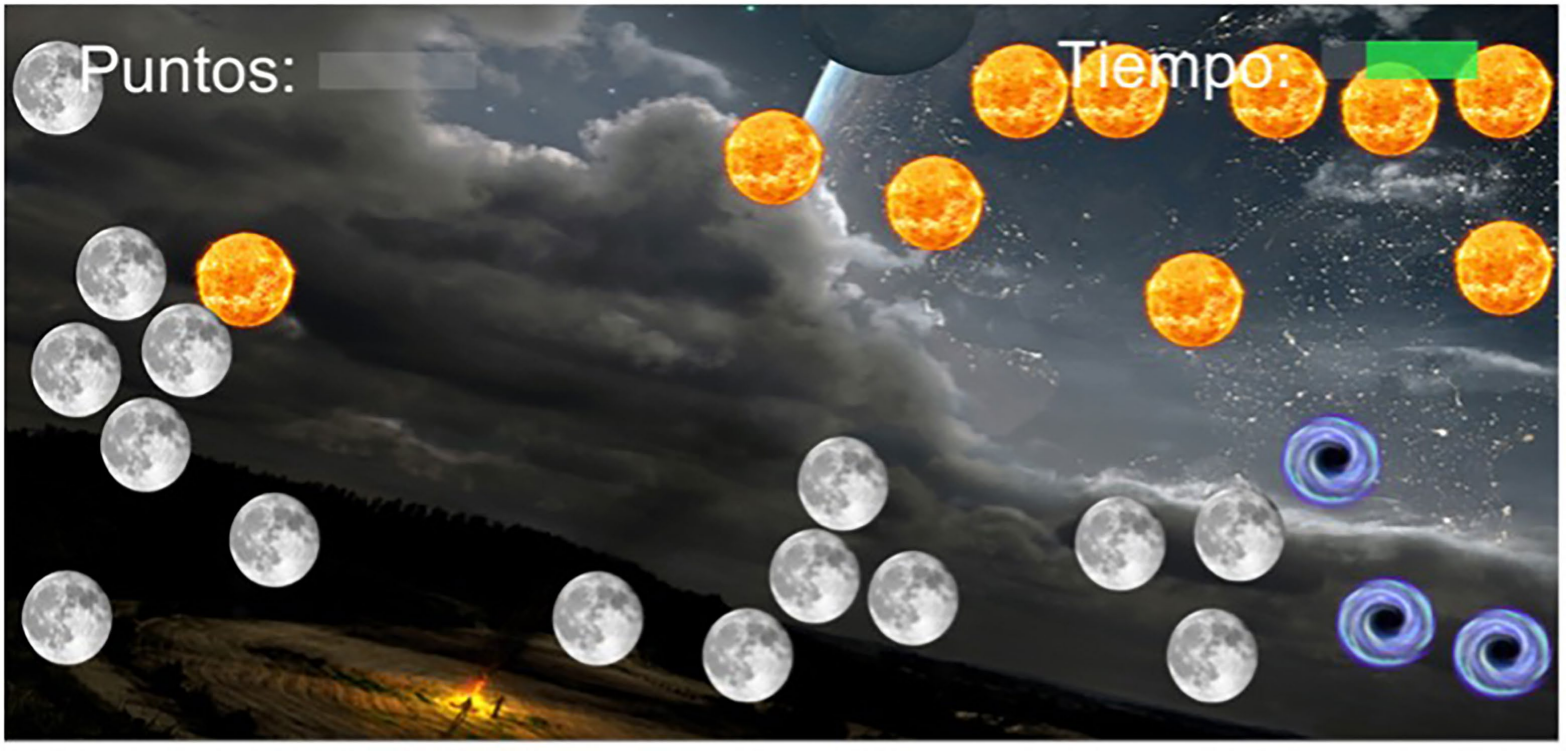
Game task.

The task to be performed in the different modes and levels is always similar. It is very simple to comply with requirement (a) above, and consists of exploding objects [in the shape of suns (5 points) and moons (1 point)], which move across the surface of the screen (see Figure 2). The way to explode the objects is by clicking on them with the mouse. The user must avoid exploding the dark holes, as in this case the user will lose 5 points. The difficulty of the tasks increases as the level increases by manipulating a series of parameters, such as the number and type of elements that appear on the screen, their size, the speed at which they move, etc. This task has been chosen to be simple enough so that virtually anyone has the necessary skills to be able to perform it, as required by Santacreu et al. (2004) of an objective test to measure personality traits. On the other hand, it is a task where it is not easy for the user to keep track of the results obtained, that is, to be able to accurately assess the quality of their performance. This is useful because it will allow us to manipulate these results and evaluate the user’s reactions to them.

At any time, the user can consult the game classification that includes two ranking types:

- Ranking of scores, where the players will appear in order from the highest to the lowest accumulated score.
- Ranking of players who have reached level 5 in each mode of the game.

We have created a software system whose architecture is composed of the following elements: Game, MetricGame, Database and two web services, one in charge of registering the data sent from the game to the database, and the other in charge of receiving queries from the game, retrieving the information from the database, and returning the response to the game. A server hosts the database and the web services. The Game component corresponds to the MT-GOT game, which includes all the defined tasks that the user must face. It interacts with the database through two web services. In this way, the game can send and register all the data generated by the participants in the database and keeps up to date the scores of the players as they are produced. The MetricGame component is responsible for automatically calculating the motivational traits of each participant once the interaction with the MT-GOT is finished. It interacts directly with the database, extracting all the necessary records of the games.

#### Training mode

The objective is to familiarize the user with the operation of the game. The user can choose between two types of training: by trying to meet a series of objectives proposed by the system, or by challenging themselves, each time trying to beat the last score obtained.

In the Training Mode, unlike the other modes, no rewards will be offered for completing the 5 levels, as it is not considered a scoring mode.

#### Betting mode

In this mode, the user will be asked to place bets related to their own performance, before facing the completion of the tasks.

#### Competitive mode

In this mode, the user can challenge someone to play while receiving challenges from other players. The two opponents will face the same situation in parallel, and the winner will be the one who achieves the best score.

One of the objectives of this mode is to try to determine the criteria applied in the selection of opponents. To do this, all users will be challenged at some point by the player who appears at the top of the ranking and by the player who appears at the bottom of the ranking. When the user receives a challenge from another player, they can choose to accept or reject it.

After each competition, if the user has lost, they will have the possibility to request a rematch to try to beat the opponent in a new game.

In both competitive and cooperative modes, whenever a user plays against or with another player, at the end of the game, they will have the opportunity to rate the performance of their opponent/partner and also to rate themselves.

When choosing a partner or opponent, the user will receive a series of statistics or results about the candidates, including the scores with which the other players have rated them in previous games in which they played together or against each other.

#### Cooperative mode

In this mode, the user can invite other players to play together and can also receive invitations from other players. The two players of a cooperative team will face the same situation together, so they will combine their efforts to explode the bubbles that appear, and both will receive the total points and coins accumulated.

For each game or level in cooperative mode, the system will propose different teammates to the user, with the aim of trying to determine the criteria applied in the selection of teammates. In the list of candidates there will be players with good and bad positions in the ranking, and when appropriate players who have previously demonstrated a critical or flattering attitude toward the user (through the ratings made at the end of each game).

#### Challenge mode

In this mode, in each level the system will propose a challenge to the user. The available challenges are cataloged by difficulty level. If the user fails the challenge proposed in a level, they will lose points and will go back to the lower level. Like in other modes, it is necessary to complete three levels to pass this mode, while the two last levels are optional.

### Assessment metric

A metric has been defined that allows quantifying the subscales taken from MTQ-LONG from the user behaviors recorded by the system. The implemented metric is detailed in Table 1.

**Table 1:** MT-GOT metric.

Determination (D)		Other referenced goals (ORG)	
Weight of each action	Actions performed by the user to be evaluated	Weight of each action	Actions performed by the user to be evaluated
10%	At the beginning of the game, the user is shown a tutorial with different explanations of how the game works. In addition, the user can access this tutorial throughout the game, either because it has been skipped at the beginning of the game or because they want to refer to it again Check whether the user follows all the tutorial steps or skips them. Calculate the percentage of the tutorial that has been completed	15%	Whenever the user finishes a game in Competitive mode, they can consult their position in the general ranking to decide whether to publish the score or not Calculate the proportion of Competitive mode games in which the user decided to look at the ranking before publishing the score
10%	Count if the user reached level 5 in all game modes. Add 2% each time a game mode is completed	15%	Whenever the user plays in Competitive or Cooperative modes, they can compare with other players (there is a button that allows to display more information about the player) before choosing the opponent or partner Calculate the proportion of times the user, before choosing partners or rivals, decides to compare with the proposed players
10%	Count if the user appears in the first, second, and third places in the competitive ranking. Add 3.33% for each position	15%	Check whether the user publishes any results in the public ranking: 1. If the result is not published, analyze whether the results are good or bad and whether the user has seen the ranking or not - If the user decided not to see the ranking, infer that the user does not care what others think - If the result is good and the user has seen the ranking, infer that the user does not care what others think - If the result is bad, infer that the user cares about other’s opinions 2. If the user decides to publish the results in the ranking, different situations should be considered: - If the user always publishes the results independently of how good they are, infer that the user does not care what others think - If the user publishes only after comparing the result with the rest, infer that the user cares what others think
10%	Count if the user appears in the first, second, and third places in the coin ranking (3.33% for each place)	10%	At the end of a game in Competitive or Cooperative mode, the user can choose to see the score given by the opponent or partner Calculate the proportion of times the user chooses to see the score given by the opponent/partner at the end of a game
20%	Whenever the user loses a game, they can play a rematch against the same opponent Calculate the percentage of times the user decides to play a rematch after losing a game against another player	5%	Observe if there is a relationship between the users invited to play and the opinions they have about the user. To evaluate this point, the user has had to visualize the score that these users have given Calculate the proportion of times the user chooses as partners those who have the best opinion of the user
20%	In the Challenge mode, whenever the user does not successfully complete a challenge, the user loses a Challenge mode level Count the number of levels the user has lost in this mode. Add 4% for each lost level	5%	Observe whether there is a relationship between the players chosen to be challenged and the opinions they have about the user. To evaluate this point, the user has had to visualize the score that these users have given Calculate the proportion of times that the user challenges those who think is a bad player
20%	In the Training, Competitive, Cooperative and Gamble game modes, count the number of games lost from level 3. Add 2% for each lost game	20%	Once the user decides whether to publish the results in the ranking, they will be asked to rate their own performance. To extract information, the user must choose to view the ranking before publishing the results When it comes to informing the user about the results, there are two options: - The results are true and correspond to their actual performance - The results are not true and appear to be much worse than they really are Evaluate whether the self-assigned score depends on the position in the ranking [it is good if the user finds to be on the top (top 5) or it is bad if the position in the ranking is bad (bottom 5)]. If this happens, it can be inferred that the self-assessment depends on a comparison with the other users
Mastery goals (MG)		15%	In Competitive mode, analyze the scores self-assigned after winning games and after losing games, and see whether there is any relationship If the user’s judgment is affected by the outcome of the game and not by the real performance, add up to 15%
Weight of each action	Actions performed by the user to be evaluated	Failure avoidance (FA)	
30%	In the Training mode, there are two types of training, one in which the system sets the objectives and the other in which the user chooses to challenge themselves trying to improve in each game. If the user chooses the second way, it is considered an indicator that the user likes to set goals to improve their performance, and 30% is added. If the user chooses the first path, the degree to which the user sets improvement goals is considered lower, and 15% is added	Weight of each action	Actions performed by the user to be evaluated
25%	Whenever the user loses a game, they can play a rematch against the same opponent Count the percentage of times the user decides to play a rematch after losing a game against another player	10%	In the Challenge mode, the user must choose whether to continue advancing levels despite the risk of losing the levels obtained, or to stay at level 3. If, once level 3 is obtained, the user does not play any more games, add 10%; if the user plays 1 more game, add 6.66%; if the user plays 2 more games, add 3.33%; and if the user plays more than 2 games, add 0%
25%	Count the proportion of situations in which the user wanted to complete all possible tasks in the game, even without being forced to do so Situations of this type are those in which the user can either complete the modes at the maximum level (5) or settle for the minimum level required (3). 5% will be added for every mode in which the user reached level 4	5%	In the Betting mode, the degree of risk in the bets placed by the user is measured by the number of coins bet and the points to be obtained. If the user bets few coins or bets to get very low scores, add 5%
10%	Count the number of times the user voluntarily repeats a game to beat a previously obtained score in both the Competitive and Challenge modes. If the user repeats once, 5% will be added; if they repeat 3 times, 10% will be added	5%	If the user chooses to play against opponents who are in the top six places in the ranking, add 0%; if the user plays against opponents in the last positions of the ranking or with very low scores, add 5%
10%	Determine whether the user decided to complete the 6 challenges of the Challenge mode or was satisfied with completing the 5 required challenges	20%	There are several situations in which the user may decide to continue playing in the mode or leave: - In the Challenge mode, the user may take the risk of losing levels after failing in the game - In the Betting mode, if the user makes a bet and fails, there is the option to try again or not - In the Competitive mode, when the user challenges someone and loses, they can challenge them again (revenge) or try to challenge someone easier to win - In the Cooperative mode, if the user does not get the minimum score with a partner, they can try again with the same partner, with a better partner, or refuse to try again - In the Training mode, if the user does not pass a level, they can try again or not, or change the type of training Once the results of all these situations have been obtained, make an average of the situations in which the user took a risk without any obligation
Competition search (CS)		10%	The user will be challenged to play against a better user before reaching level 3. If the user does not accept, add 10%
Weight of each action	Actions performed by the user to be evaluated	10%	Another user will ask for help with the game (information that should be known because it is relatively simple). If the user does not give the information, being prone to help on other occasions, it can be inferred that the user does not want to risk giving a wrong answer, then add 10%
33%	Compare the number of games the user has played in Competitive mode versus Cooperative mode. If the number of Competitive games is more than double the number of Cooperative games, add 33%	10%	Analyze the number of games that the user has decided to play in each degree of difficulty, out of the total number of games played. If the user has played easy challenges, add 10%; if the user has played a medium difficulty challenge, add 5%; and if the user has played a difficult challenge, add 0%
16,5%	Obtaining better results in Competitive mode versus other modes may reflect a tendency to feel comfortable in competition. If the highest score was obtained in this mode, add 16.5%	10%	If the user has not requested much help, we may infer that they prefer the complicated path of discovering how the game works by themselves. In contrast, requesting a lot of information in detail before facing a task implies a tendency to avoid failure If the user completes the tutorial and asks for help from the virtual environment bot before facing the games, add up to 10%
16,5%	The degree to which the user makes more attempts in Competitive mode than in Cooperative mode, to overcome an unsuccessful attempt, may reflect their degree of competitiveness. If the number of games is twice the number of attempts in Cooperative mode, add 16.5%	3%	If the user prefers not to look at any score received from rivals or peers, it may reflect a tendency to avoid being evaluated. Add 3%
33%	If the user chooses Competitive mode earlier than Cooperative mode, add 33%	5%	If the user does not publish their results in any ranking or visits them, it may reflect a tendency to avoid being evaluated. Add 5%
Desire to learn (DL)		12%	When reaching level 3 in cooperative mode, the user has the option to disable external evaluations. If the user deactivates the external evaluations, add up to 12%
Weight of each action	Actions performed by the user to be evaluated		
30%	The degree of completeness with which the user has explored the tutorial in normal mode will be measured, since the user has the option to leave the different blocks of information at any time Calculate the percentage of the tutorial that has been completed		
15%	The degree of completeness with which the user has explored the optional additional material within the tutorial will be measured Calculate the percentage of additional material that has been explored		
15%	The path followed by the user in the environment, and the degree to which the user has explored the entire environment and the objects within the environment not directly related to the game, will be determined Calculate the percentage of map exploration		
20%	In the Training mode, there are two types of training, one in which the system sets the objectives and the other in which the user chooses to challenge himself trying to improve in each game. If the user chooses the latter way, the degree of real improvement observed in the user’s performance throughout the training will be determined, as an indicator of effort to learn from experience and do better and better. (compare the score obtained by the user in each game of the Training mode and see if he has to repeat a game because he got a worse score)		
20%	To determine the degree of improvement observed in the user’s performance in the whole game over time, we will compare the score obtained in all game modes (except Training) in the first game (level 0) and in the last game (level 3 or 5) Add 5% for each game mode in which there was improvement		

All percentages are approximate and rounded up to 100% for each subscale. In some cases, since each participant freely chooses the actions to perform, certain situations considered in the metric may not occur. In this case, the metric is automatically adjusted, adapting the weights according to the situations that the participant has actually experienced in the game.

## Validation of the objective behavioral test for the assessment of motivational traits

Once the MT-GOT objective test system was implemented, an experiment with volunteers was carried out to collect sufficient data to validate the performance of MT-GOT as an objective test to assess motivational traits.

### Materials

To carry out the experiment, a computer room was used with five computers with Internet access and a WebGL compatible browser (Google Chrome) installed. It was verified in advance that the MT-GOT game ran correctly on the computers in the computer room, as well as the communication between the MT-GOT and the server where the database is hosted.

Two questionnaires were implemented in Google Forms, corresponding to a demographic survey and the MTQ-LONG questionnaire. An informed consent form was also prepared for participation in the experiment, explaining to the participants what the experiment consists of and how their personal data would be treated. Finally, an information sheet was created, with information about the game (mechanics and objective of the game) to be used as an aid while playing.

### Method

The participants were 31 adults (23 men and 8 women) related to the Universidad Politécnica de Madrid, some as students and others as faculty members. The average age was 27 years (ranging from 18 to 40 years). Recruitment was carried out by publishing an advertisement in student forums and by invitations to faculty colleagues. No incentives were offered to the participants.

The participants carried out the experiment in batches of five people. The procedure was as follows: first, all participants read and signed the informed consent about the experiment, and then completed a brief demographic survey.

Half of the participants (16) first completed the MTQ-LONG questionnaire and then played the MT-GOT, and the other half (15) first played the MT-GOT and then completed the MTQ-LONG questionnaire. Before starting with the MT-GOT, participants received the information sheet with game information to read before playing.

### Statistical analysis

The statistical software used to perform the analysis of the data collected in this experiment was Minitab 19 (2020) and G*Power (2020). Four statistical analyses were performed:

- The first step aimed to analyze the normality of the data distributions. For the normality test we applied the Anderson-Darling statistic because it tends to be more effective in detecting departures from normality in the tails of the distribution.
- The second study analyzed the correlation, for each dimension, between the values calculated by MTQ-LONG and MT-GOT. In the correlation study we applied Pearson’s statistic for samples following a normal distribution and Spearman’s statistic for samples following a non-normal distribution.
- The third study focused on the analysis of the statistical power of correlations.
- We concluded with a study of interrater agreement between MTQ AND MT-GOT, including the percentage of agreement among raters, Cohen’s Kappa (Cohen, 1960) and Kendall’s coefficient of concordance (Legendre, 2010) for ordinal outcomes. The F test was performed based on Kendall’s coefficient of concordance to test against the null hypothesis that the ratings of different raters are not concordant with each other. A *p* value of <=0.05 was considered statistically significant and the null hypothesis was rejected. The agreement between MTQ and MT-GOT was compared by a 95% confidence interval (CI) for the difference in Cohen’s kappa or Kendall’s coefficient of concordance statistic. The interpretation of the Kappa coefficient and Kendall’s coefficient of concordance values has been based on the following intervals: poor (0), slight (0.01–0.2), fair (0.21–0.4), moderate (0.41–0.6), substantial (0.61–0.8), and almost perfect (0.81–1) (Landis and Koch, 1977; Viera and Garrett, 2005).

For the inter-rater agreement study, we decided to transform the data to an ordinal scale in order to classify the users into five grades (1, 2, 3, 4, and 5) which would be equivalent to (Very low, low, medium, high, and very high) within each dimension of the motivational traits. This transformation allows us to better interpret the meaning of the results of each participant in each dimension, allowing us to observe more clearly when a person stands out or lacks a specific motivational trait, without the need to consider the maximum score, the minimum score, and the range of possible values in each dimension. The degree of agreement between the ratings provided by MTQ and MT-GOT will be based on the ordinal transformed scales. We have performed this transformation by dividing the range of possible MTQ and MT-GOT scores (which are the same for each dimension) into five equally-sized intervals.

## Results

Throughout the experiment, data were collected from 1,256 games played, an average of 40 games per participant. Each game lasted an average of 20 s, which is equivalent to an average playing time of 13 min and 20 s. The participants placed 221 bets, an average of 7 bets per participant, and 652 levels were passed out of the possible 806. The database is available upon request from the corresponding author.

The game implements the MT-GOT metric and provides as an output the value computed for each motivational trait, in the same format as the one provided by the MTQ questionnaire. No further preprocessing was necessary for the analysis of the results other than the transformation into an ordinal scale for the inter-rater agreement study.

The results obtained with the two methods of evaluation of motivational traits for the 31 participants are shown in Table 2.

**Table 2:** Motivational traits values.

ID group	ID participant	D-MTQ	D- MT-GOT	DL-MTQ	DL- MT-GOT	MG-MTQ	MG- MT-GOT	ORG-MTQ	ORG- MT-GOT	CS-MTQ	CS- MT-GOT	FA-MTQ	FA- MT-GOT
1	1	20	25	20	30	34	30	12	20	−18	−10	19	25
1	2	19	10	15	20	35	43	29	30	−1	−5	17	15
1	3	18	22	19	25	29	38	18	23	8	0	12	16
1	4	30	26	27	25	38	38	27	25	11	12	23	42
2	5	22	26	14	18	35	32	31	36	0	1	24	20
2	6	25	29	29	19	38	36	38	36	9	−2	19	23
2	7	12	16	20	33	28	33	32	38	1	2	38	43
2	8	25	30	24	26	34	32	49	40	17	14	19	13
2	9	27	16	31	26	40	21	36	36	−4	−1	25	33
2	10	21	21	30	27	39	33	32	30	17	2	19	23
2	11	13	31	14	21	34	40	24	30	14	16	34	32
2	12	18	30	26	29	33	31	36	31	2	3	50	13
1	13	17	15	23	25	34	36	30	29	−5	0	27	29
1	14	22	21	22	26	38	39	36	31	13	6	29	24
1	15	11	10	3	9	21	26	27	30	−16	0	27	36
1	16	16	20	18	25	27	30	41	35	5	3	41	50
1	17	13	23	14	26	31	41	29	41	0	16	32	15
1	18	19	10	13	23	31	23	18	33	−11	−2	38	36
1	19	13	21	17	18	46	41	30	31	23	13	13	16
1	20	19	23	19	23	37	40	35	33	10	13	33	23
2	21	21	24	17	23	33	37	24	38	0	13	27	25
2	22	20	25	23	30	34	32	22	23	−6	−2	24	31
2	23	23	16	19	15	37	22	31	19	0	−2	38	27
2	24	23	21	34	15	41	29	18	22	−6	−1	30	21
1	25	21	23	24	21	38	40	24	30	−7	2	38	25
1	26	23	24	21	21	47	26	23	23	−7	−9	46	50
2	27	16	21	11	19	22	24	31	25	6	2	42	27
2	28	19	22	25	17	41	26	37	31	0	−2	42	35
2	29	31	29	40	33	50	31	13	26	−12	−14	6	20
1	30	31	34	32	29	46	33	20	26	0	−1	24	21
1	31	22	26	27	31	35	31	5	23	−6	−5	12	16

### Differences in means

From the motivational traits data, we have performed a first statistical analysis to explore the difference in means for each dimension, which can be seen in Table 3. The difference in means is low, below 3 points in all dimensions.

**Table 3:** Differences in means and average error.

Subscale	Average MTQ scores	Average MT-GOT scores	Difference in means	Interval	% error
Determination (D)	20.32	22.26	−1.935	32 to−18	−3.87%
Desire to learn (DS)	21.65	23.48	−1.839	40 to−5	4.09%
Mastery goals (MG)	35.68	32.71	2.968	52 to−3	5.40%
Other referenced goals (ORG)	27.68	29.81	−2.129	64 to −1	−3.28%
Competition seeking (CS)	1.19	2	−0.806	26 to −19	1.79%
Failure avoidance (FA)	28	26.61	1.387	71 to 6	1.80%

To assess the importance of these differences in means within each dimension, it is necessary to relate them to the value intervals that can be obtained for each dimension. We observe that the differences between the mean values obtained with the MTQ and the MT-GOT are less than 5.5% of the range of each interval. If we look at Table 3, the largest difference is 2,968 points in the Mastery Goals dimension, where the interval varies between (52 and 53), i.e., producing a range of 55 total points. Therefore, the game has an average error of 5.45% on those 55 total points. The smallest difference is 0.806 points and occurs in the Competition Seeking dimension, where the interval varies between 26 and-19 (45 total points), and therefore, MT-GOT has an average error of-1.7% on those 45 total points. We can see the rest of the results in Table 3.

We can observe that in some dimensions MT-GOT seems to obtain higher valuations than MTQ, while in other dimensions there is a trend to undervaluation with respect to MTQ. To correct, as far as possible, the tendency that MT-GOT may have to overvalue or undervalue certain dimensions compared to MTQ, we decided to carry out a transformation of the results provided by MT-GOT, which consists of adding the difference in means obtained in each dimension to all the results of every participant. From now on, we will work with these transformed data.

### Normality analysis

First, we tested the normality of the results obtained by both methods of assessing motivational traits (MTQ and MT-GOT). Table 4 displays the results of the application of the Anderson-Darling statistic.

**Table 4:** Normality test.

Subscale	AD	df	Sig.
D-MTQ	0.358	31	0.431
D-MT-GOT	0.561	31	0.135
DL-MTQ	0.163	31	0.938
DL- MT-GOT	0.247	31	0.734
MG-MTQ	0.427	31	0.294
MG- MT-GOT	0.412	31	0.320
ORG-MTQ	0.275	31	0.635
ORG- MT-GOT	0.408	31	0.327
CS-MTQ	0.316	31	0.524
CS- MT-GOT	1.118	31	0.005
FA-MTQ	0.243	31	0.747
FA- MT-GOT	0.628	31	0.093

From these data we can conclude that the CS-MT-GOT (Competition Seeking) dimension does not follow a normal distribution, since its value of p is clearly lower than 0.05. For the FA-MT-GOT (Failure Avoidance) dimension, since its value of p is 0.093 (bigger than but close to 0.05), we cannot affirm that the sample is not normal, but for the correlation test we will apply both Pearson’s statistic (for samples that follow a normal distribution) and Spearman’s statistic (for samples that follow a non-normal distribution). The Minitab statistical software also warns us of the possibility that a sample that appears to be normal is not, and vice versa, due to the small number of samples to be evaluated.

### Correlation analysis

Second, we performed a correlation test between the values obtained with the MTQ and the MT-GOT, to see if there is a linear relationship between the values provided by each method. All correlations have been evaluated with a 95% confidence level.

#### Determination dimension (D_MTQ vs. D_MT-GOT)

**Figure 3:**
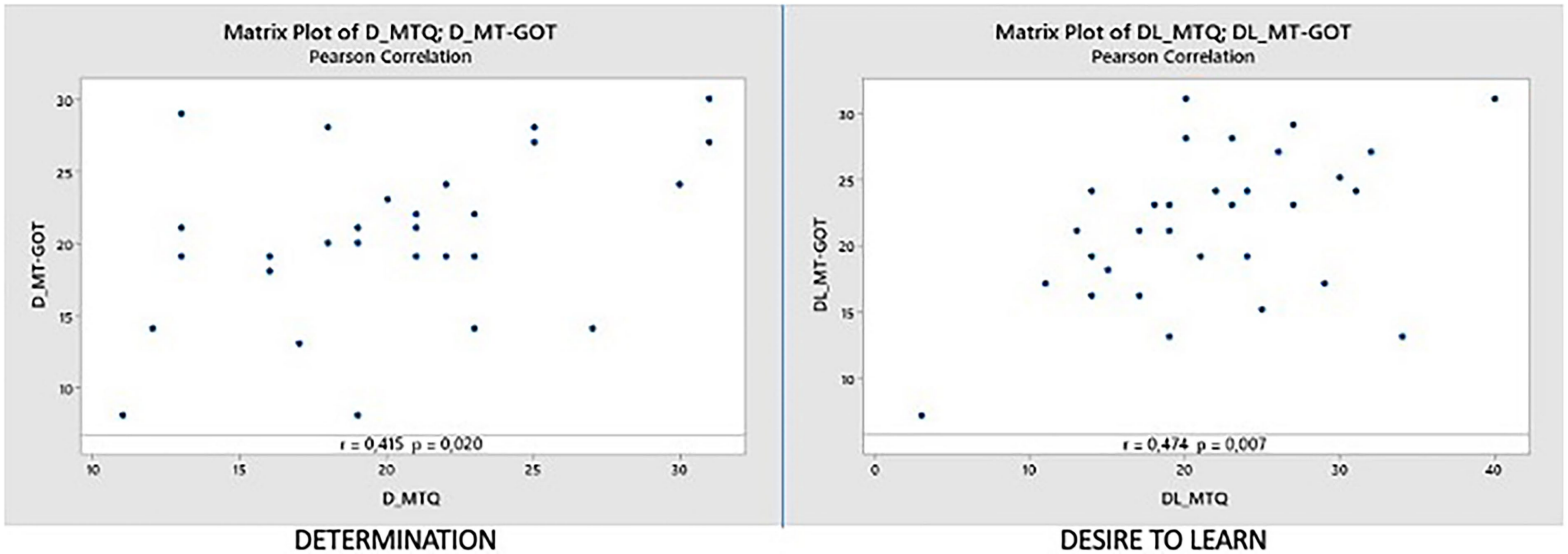
Correlations D_MTQ vs. D_MT-GOT and DL_MTQ vs. DL_MT-GOT.

In this dimension we have a Pearson correlation coefficient of 0.415, and we can observe that there is a lot of dispersion between the points of the graph. The value of *p* is 0.020, indicating that the relationship, although not very high, is statistically significant at the α = 0.05. The correlation has a positive direction (as we can see in Figure 3 left). Several outliers stand out in the graph, for example, participant 9 (27,14.1), participant 11 (13,29.1), and participant 18 (19,8.1). These points will be analyzed later.

#### Desire to learn dimension (Dl_MTQ vs. Dl_MT-GOT)

In this dimension we have a degree of correlation of 0.474, and we can observe that there is a lot of dispersion between the points in the central area of the graph. The value of p is 0.007, which indicates that the relationship is statistically significant at the level of α = 0.05. The relationship has a positive direction (as we can see in Figure 3 right). We found a number of outliers in the center of the graph, for example, participant 24 (34.13.2), participant 6 (29.17.2) and participant 7 (20.31.2). These points will be analyzed later.

#### Mastery goals dimension (MG_MTQ vs. MG_MT-GOT)

**Figure 4:**
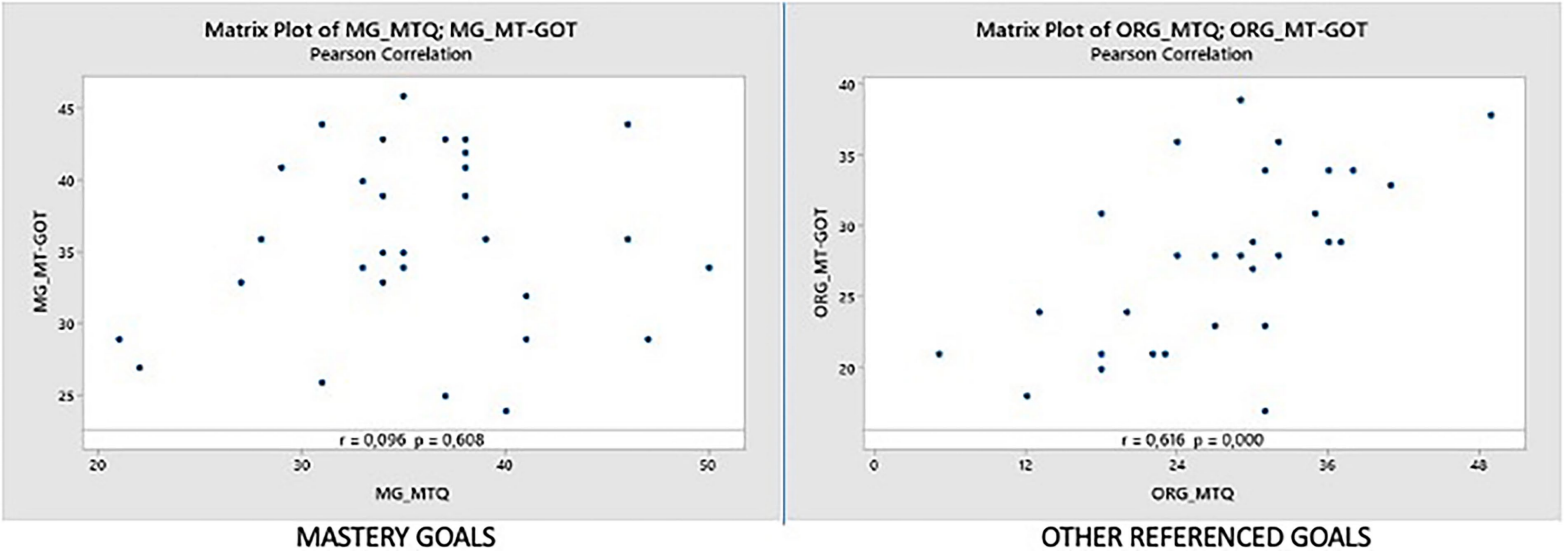
Correlations MG_MTQ vs. MG_MT-GOT and ORG_MTQ vs. ORG_MT-GOT.

In this dimension we have an almost non-existent degree of correlation since the Pearson correlation coefficient is 0.096 (as we can see in Figure 4 left). However, the value of p is 0.608, indicating that the relationship is not statistically significant at the level of α = 0.05. We will analyze this dimension in more detail later.

#### Other referenced goals dimension (ORG_MTQ vs. ORG_MT-GOT)

In this dimension we have a Pearson correlation coefficient of 0.616, and we can observe that there is not much dispersion between the points on the graph. The value of p is 0.000, indicating that the relationship is statistically significant at the level of α = 0.05. The plot has a positive direction (as we can see in Figure 4 right) and is quite linear. We found a number of outliers in the graph, for example, participant 31 (5,20.9), participant 23 (31,16.9), and participant 18 (18,30.9). These points will be analyzed later.

#### Competition seeking dimension (CS_MTQ vs. CS_MT-GOT)

**Figure 5:**
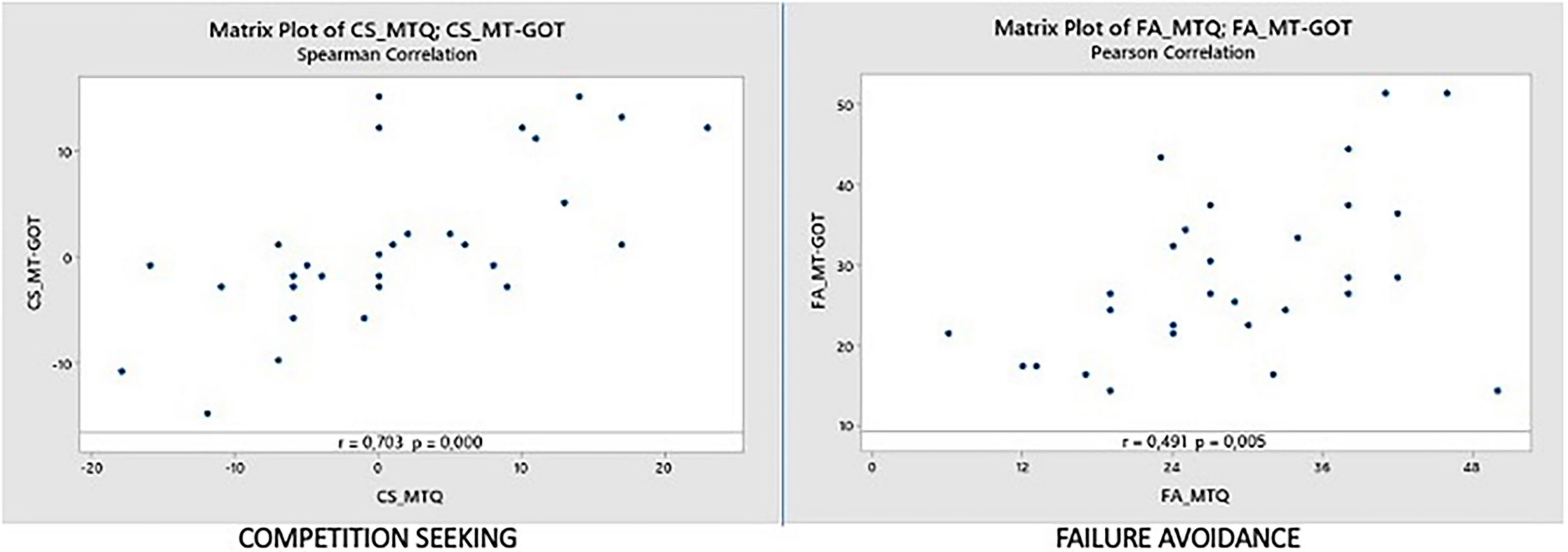
Correlations CS_MTQ vs. CS_MT-GOT and FA_MTQ vs. FA_MT-GOT.

In this dimension we have a Spearman correlation coefficient of 0.703. This coefficient is the highest of all subscales; therefore, we can state that it is the dimension where the results of the MTQ and MT-GOT assessment correlate best and have the strongest linear relationship. The value of *p* is 0.000, indicating that the relationship is statistically significant at the level of α = 0.05. The graph has a positive direction (as we can see in Figure 5 left). The main outliers are participant 10 (17,1.2), participant 15 (−16,-0.8), and participant 17 (0,15.2). These points will be discussed later.

#### Failure avoidance dimension (FA_MTQ vs. FA_MT-GOT)

In this dimension, we have a Pearson correlation coefficient of 0.91 and a Spearman correlation coefficient of 0.504. The *p* values are 0.005 and 0.004 respectively, indicating that the relationships are statistically significant at the α = 0.05. The values are similar for both tests and close to the 0.5 value. The plot has a positive direction (as we can see in Figure 5 right). We found a number of outliers in the graph, for example, participant 12 (50,14.4), participant 4 (23,43.4) and participant 17 (32,16.4). These points will be analyzed later.

### Power of correlation tests

Third, we performed a statistical analysis of the power of the correlation tests we ran before (see Table 5). This statistical test describes the probability that a test correctly identifies a real effect, since it may happen that we obtain significant results but cannot rely on them. The power of a hypothesis test is affected by the sample size, the difference, the variability of the data, and the significance level of the test.

**Table 5:** Power of correlations.

Subscale	Power (1-β err prob)
D	0.6940098
DL	0.5672584
MG	0.0200040
ORG	0.8973038
CS	0.9814913
FA	0.6491792

The results obtained are positive on most subscales, except in the MG dimension, where the correlation results already showed a value close to zero.

The powers are very good in the CS, ORG, and D subscales and slightly worse in FA and DL.

### Inter-rater agreement

Lastly, we performed, individually by dimension and in general with all the evaluations made by the MTQ and MT-GOT, an analysis of inter-rater agreement.

With Cohen’s kappa coefficient test and Kendall’s concordance coefficient, we can quantify the effect of chance on the degree of agreement observed between the two methods for assessing motivational traits. All the results can be seen in Table 6. The values in the tables are rounded to two decimal places.

**Table 6:** Cohen’s kappa and kendall’s coefficients.

Subscale	Inspected	Matched	Cohen’s Kappa	Value of *p*	Kendall’s coefficient	Value of *p*
D	31	20	0.36	0.01	0.74	0.05
DL	31	18	0.33	0.00	0.73	0.05
MG	31	14	0.031	0.40	0.54	0.36
ORG	31	19	0.30	0.02	0.76	0.03
CS	31	15	0.30	0.00	0.82	0.02
FA	31	18	0.38	0.00	0.71	0.06
General	186	104	0.41	0	0.87	0

The results obtained are very satisfactory. Most notably, considering all the MTQ and MT-GOT assessments for each participant, we obtain a Cohen’s Kappa coefficient of 0.41, which is equivalent to moderate agreement, and a Kendall’s coefficient of 0.87, which is interpreted as almost perfect agreement. The *p* values are 0, indicating that the relationships are statistically significant at the α = 0.05.

Of the total of 186 evaluations performed (6 dimensions for 31 participants), 104 agree, which corresponds to 56% of the total. The MG dimension obtains the worst agreement results between the MTQ and MT-GOT evaluators, which is in line with the results obtained in the correlation analysis. These results require us to reflect deeply on what is happening in this dimension.

## Discussion

In this section, we analyze the results obtained from the experiment, the differences observed, and the possible reasons for these discrepancies between the two methods of assessing motivational traits.

The most important points that deserve a more detailed analysis are the outliers in the correlation study between the MTQ and MT-GOT subscales, the poor result obtained in the statistical analyses on the Mastery Goals (MG) dimension, and the good results obtained in general on the rest of the subscales.

### Outliers in correlations

In this section, we analyze the three most outstanding outliers for each subscale (those with a greater distance between the MTQ and MT-GOT evaluations) and we determine the reason for this difference between the MTQ and MT-GOT evaluations. First, the Determination subscale is analyzed. Table 7 shows the three outliers that have been selected. The score obtained from the MTQ and the MTQ-GOT and their corresponding rounded percentages can be observed within the subscale. To understand these differences, the table describes the user’s expected behavior in the MT-GOT test according to the score obtained, and the actual behavior recorded in the MT-GOT. The same process has been followed for each dimension.

**Table 7:** Outliers in determination.

ID participant	MTQ value	MT-GOT value	Expected behavior MT-GOT	Real behavior MT-GOT
9	27 (90%)	14.1 (64%)	The user would make 9 failed attempts after completing level 3 in the Training, Competitive, Cooperative and Betting game modes (18% out of 20%) The user would obtain level 5 in 4–5 game modes. (8–10% out of 10%)	The user makes 4 unsuccessful attempts after completing level 3 in the Training, Competitive, Cooperative, and Betting game modes (8% out of 20%) The user obtains level 5 in 0 game modes (0% out of 10%)
11	13 (62%)	29.1 (94%)	The user would make 4 failed attempts after completing level 3 in the Training, Competitive, Cooperative and Betting game modes (11% out of 20%) The user would obtain level 5 in 3 game modes (6% out of 10%) The user would lose 3 levels in Challenge mode (12% out of 20%)	The user makes 11 failed attempts after completing level 3 in Training, Competitive, Cooperative, and Betting game modes (20% out of 20%) The user obtains level 5 in 5 game modes (10% out of 10%) The user loses 5 levels in Challenge mode (20% out of 20%)
18	19 (72%)	9.1 (54.%)	The user would make 7 failed attempts after completing level 3 in the Training, Competitive, Cooperative and Betting game modes (14% out of 20%) The user would obtain level 5 in 3–4 game modes (6–8% out of 10%) The user would lose 3–4 levels in Challenge mode (12–16% out of 20%)	The user makes 3 failed attempts after completing level 3 in the Training, Competitive, Cooperative and Betting game modes (6% out of 20%) The user obtains level 5 in 0 game modes (0% out of 10%) The user loses 3 levels in Challenge mode (12% out of 20%)

The Mastery Goals (MG) dimension is the dimension in which we obtained the worst results. The mean difference with the MTQ (2.968) represents the highest percentage of error (5.40%). If we observe the MTQ and MT-GOT scores for this subscale, in Table 2, the results are not uniform, since in the evaluation of many users, the MT-GOT score is well above or below the MTQ score. This phenomenon occurs in half of the participants in the experiment. The most outstanding cases are those of participants 9, 17, 26, and 29.

Analysis of these tables demonstrates that, sometimes, there is a great difference between how people perceive themselves and how they actually act. This phenomenon, when trying to correlate the two sources of data (questionnaires and objective behavioral tests), has already been reported in the literature (Skinner and Howart, 1973; Cattell and Kline, 1977). Our hypothesis is that a higher degree of confidence should be given to the behavior demonstrated by a person in a concrete situation presented to them in a real way as opposed to the behavior reported by the same person in an analogous fictitious situation, in the case of discrepancies as strong as the ones presented here. At least, we should not discard the scores provided by MT-GOT as completely wrong, just because there is not a perfect match with MTQ.

However, it could happen that the situations described in the MTQ have not been accurately reproduced, or that the users have not been able to assess them in the same way. Since the motivational diagnostic test is a game, it is possible that people do not perceive situations with the same severity or with the same risk associated with making a mistake.

Another possibility is that the MT-GOT metric is not totally well adjusted in the weights associated with the behaviors that are most decisive for each subscale.

It can also be observed that there are several participants who appear repeatedly as outliers, such as participants 9 (2 appearances), 18 (2 appearances), 17 (3 appearances), and 23 (2 appearances). This fact suggests that perhaps the specific profile of these participants makes the MT-GOT evaluation not work well in them. In these four participants, a specific profile has been observed in which the participant’s self-perception in the different subscales tends to be higher, as would be the case of participant 9 and 23; on the other hand, participant 17 has a lower self-perception. No clear trend can be observed for participant 18 (see Table 8).

**Table 8:** Difference between MTQ-GOT and MTQ SCORES (by participant).

Id participant	Error D	Error DL	Error MG	Error ORG	Error CS	Error FA	Total error	% error (over 342 points)
1	−5	−10	4	−8	−8	−6	41	11,99
2	9	−5	−8	−1	4	2	29	8,48
3	−4	−6	−9	−5	8	−4	36	10,53
4	4	2	0	2	−1	−19	28	8,19
5	−4	−4	3	−5	−1	4	21	6,14
6	−4	10	2	2	11	−4	33	9,65
7	−4	−13	−5	−6	−1	−5	34	9,94
8	−5	−2	2	9	3	6	27	7,89
**9**	**11**	**5**	**19**	**0**	**−3**	**−8**	**46**	**13,45**
10	0	3	6	2	15	−4	30	8,77
11	−18	−7	−6	−6	−2	2	41	11,99
12	−12	−3	2	5	−1	37	60	**17,54**
13	2	−2	−2	1	−5	−2	14	4,09
14	1	−4	−1	5	7	5	23	6,73
15	1	−6	−5	−3	−16	−9	40	11,7
16	−4	−7	−3	6	2	−9	31	9,06
**17**	**−10**	**−12**	**−10**	**−12**	**−16**	**17**	**77**	**22,51**
**18**	**9**	**−10**	**8**	**−15**	**−9**	**2**	**53**	**15,5**
19	−8	−1	5	−1	10	−3	28	8,19
20	−4	−4	−3	2	−3	10	26	7,6
21	−3	−6	−4	−14	−13	2	42	12,28
22	−5	−7	2	−1	−4	−7	26	7,6
**23**	**7**	**4**	**15**	**12**	**2**	**11**	**51**	**14,91**
24	2	19	12	−4	−5	9	51	**14,91**
25	−2	3	−2	−6	−9	13	35	10,23
26	−1	0	21	0	2	−4	28	8,19
27	−5	−8	−2	6	4	15	40	11,7
28	−3	8	15	6	2	7	41	11,99
29	2	7	19	−13	2	−14	57	**16,67**
30	−3	3	13	−6	1	3	29	8,48
31	−4	−4	4	−18	−1	−4	35	10,23

Id participant, participant identification number; Error D, Error in Determination Error; DL, Error in Desire to Learn; Error ORG, Error in Other Referenced Goals; Error FA, Error in Failure Avoidance; Total error, sum of errors in absolute values; and % error (over 342 points), percentage of total error over 342 (maximum possible difference).

As can be seen, there are several participants where the deviation is more pronounced than in others. The participants that stand out the most in this table are participants number 17, 12, 29 and 18, all of them accumulating an error above 15%, and close to that 15% are participants 23 and 24.

### General results and implications

This research is in line with previous research in the use of game-based assessments of personality traits (full games used to assess specific constructs), which is becoming increasingly available and popular (Weidner and Short, 2019; Wu et al., 2022). However, to our knowledge, this is the first attempt to create an objective tool to measure the motivational traits of a given person. In this sense, it is not possible to compare with other computational tools, but only directly with the questionnaire, which is a self-referred tool and, therefore, subjective. The results obtained are very satisfactory, since we have reached a great index of agreement between the two evaluation methods, with a 0.41 in the Cohen index, which is equivalent to moderate agreement, and a 0.87 in the Kendall coefficient, which is equivalent to an almost perfect agreement. And this despite the great difficulty of trying to relate the two different sources of data (questionnaire and objective test) for the reasons already stated.

In future studies, it would be necessary to continue the MT-GOT validation process with a larger number of participants. In this way, we could observe whether these equivalence trends between the subscales reappear and whether the patterns observed in this evaluation are repeated or not.

We can highlight that MT-GOT is a very enjoyable game, and the study participants continued playing once the experiment was finished voluntarily, so we could say it is a pleasant objective test for the user. Once the experiment was completed, we asked participants their opinion about MTQ and MT-GOT, and they said they had not imagined that both evaluation methods could measure the same thing. In this way, we achieved the concealment factor in the objective test, allowing participants to act naturally. Opinions about the MTQ were less positive because they considered it a very long and cumbersome questionnaire. MT-GOT is available to be shared with the scientific community (although it is currently only available in Spanish).

### Limitations and future work

It should be noted that the validation of the MT-GOT was performed with a relatively small number of participants, so we consider the positive results obtained as a first confirmation of the feasibility of automatically modelling motivational traits by an objective test. The proposed metric for the behavior-based estimation of motivational traits will continue to be refined, with the goal of increasing its reliability. We also would like to investigate the root causes of the observed discrepancies and try to determine whether and when the results of MT-GOT could be considered more or less reliable than the ones obtained *via* MTQ.

Gender bias is another threat to the validity of the results, given that only one-third of the sample were women. In future research, a more gender-balanced sample should be targeted. Furthermore, the profile of the participants in the validation was somewhat biased toward people experienced in the use of computer applications and in their youth or middle age. We are concerned that the kind of game designed could not be appropriate for older people or people with limited experience in the use of computer applications, so we have already implemented an adapted version of the objective behavioral test for older people, which presents a simpler game task and improves the usability and accessibility of the interface to cope with age-related limitations in perception and cognition.

The task proposed in the game might also not be equally motivating for all users or user groups. Even if this is not expected to have a big impact in the assessment of motivational traits, the lack of motivation in the game might occasionally lead to decisions that are different (for example, people with a competitive profile might not be so motivated to compete in that particular kind of task). An interesting line of future work should investigate how the results of game-based assessment depend on the level of motivation raised by the game.

Although the initial effort in developing the game is certainly high, and this could be considered as a limitation, it should be noted that once the game is finished, it can be reused without extra costs. Also, game-based assessment allows to perform many tests simultaneously without the specialized professional effort that is necessary to administer and assess a questionnaire. Moreover, a complete track of the user’s behavior is automatically registered by the game, being available for later inspection or additional analysis, something that is not possible with traditional questionnaires.

Even if it is feasible now to inspect the track of the user’s behavior in order to understand the rationale for the inferred user’s motivational profile, a very useful future development should tackle improving the explainability of the results, with simple-to-use visualization and explanation tools that make apparent how the MT-GOT metric was applied and which user’s actions and decisions led to the valuation for each motivational trait.

## Conclusion

MT-GOT fulfills the purpose for which it was created (objective assessment of motivational traits), and the results obtained indicate that it measures very approximately the same as the MTQ questionnaire.

We decided that a computer game would be a good approach to implement an objective behavioral test, given that playing is an inherently motivating activity that would also allow us to conceal quite easily the final goal of the system (modelling the user’s motivational traits). However, the key aspect in the design of the objective test is not really the kind of activity performed by the users, but the fact that they are faced with relevant situations where they have the option to behave in different ways. The choice of a specific kind of behavior or another is what the metric considers in the estimation of motivational traits.

The differences between the mean values obtained with the MTQ and the MT-GOT are less than 5.5% of the interval range for each dimension. Competition Seeking (CS) is the dimension where the results of the MTQ and MT-GOT evaluation correlate in the best way and have the strongest linear relationship, with generally good correlation results obtained on the rest of the subscales except for the Mastery Goals (MG) dimension, also the one with the highest percentage of error (5.40%).

The kappa and Kendall’s coefficients, quantifying the effect of chance on the degree of agreement observed between the two methods for assessing motivational traits, show that the high level of agreement between both methods is consistent, and therefore we can reliably use this software application to assess users’ motivational traits.

The Master Goals dimension obtains the worst results of agreement between the MTQ and MT-GOT evaluators. More research is needed to find the root causes of this discrepancy. It could happen that the MT-GOT metric or the game design does not adequately capture the situations posed by the MTQ. However, we should not forget that sometimes there is a great difference between how people perceive themselves and how they actually act.

To conclude, we have created an objective tool to obtain a model of the user’s motivational traits without having to rely on a questionnaire. This opens the possibility of building software applications that adapt to the user’s motivational profile, providing them with the kind of motivational support that best fits their needs. Currently, we are integrating MT-GOT into an online rehabilitation software application that, based on the motivational traits of the users obtained through MT-GOT, will be able to adapt the rehabilitation tasks and goals to the motivational traits of each patient with the aim of reducing the rate of abandonment of therapy.

## Data availability statement

The raw data supporting the conclusions of this article will be made available by the authors, without undue reservation.

## Ethics statement

The studies involving human participants were reviewed and approved by Universidad Politécnica de Madrid. The patients/participants provided their written informed consent to participate in this study.

## Author contributions

AdA and EV-M contributed to the conception and design of the research, as part of DF-A’s Ph.D. DF-A implemented the objective behavioral test, conducted the experiment, performed the statistical analysis, and wrote the first draft of the manuscript. All authors contributed to the article and approved the submitted version.

## Funding

This research was partially funded by the Spanish Ministry of Science and Innovation research grant (PID2019-108408RB-C21) – Active UP project, and by the European Project POSITIVE (reference 20683) funded by EIT Health.

## Conflict of interest

The authors declare that the research was conducted in the absence of any commercial or financial relationships that could be construed as a potential conflict of interest.

## Publisher’s note

All claims expressed in this article are solely those of the authors and do not necessarily represent those of their affiliated organizations, or those of the publisher, the editors and the reviewers. Any product that may be evaluated in this article, or claim that may be made by its manufacturer, is not guaranteed or endorsed by the publisher.
